# Evaluation of Amyotrophic Lateral Sclerosis-Induced Muscle Degeneration Using Magnetic Resonance-Based Relaxivity Contrast Imaging (RCI)

**DOI:** 10.3390/tomography7020015

**Published:** 2021-05-05

**Authors:** Sudarshan Ragunathan, Laura C. Bell, Natenael Semmineh, Ashley M. Stokes, Jeremy M. Shefner, Robert Bowser, Shafeeq Ladha, C. Chad Quarles

**Affiliations:** 1 Barrow Neuroimaging Innovation Center, Division of Neuroimaging Research, Barrow Neurological Institute, Phoenix, AZ 85013, USA; laura.bell@barrowneuro.org (L.C.B.); natenael.semmineh@Barrowneuro.org (N.S.); ashley.stokes@barrowneuro.org (A.M.S.); chad.quarles@barrowneuro.org (C.C.Q.); 2 Department of Neurology, Barrow Neurological Institute, Phoenix, AZ 85013, USA; Jeremy.Shefner@DignityHealth.org (J.M.S.); Robert.Bowser@DignityHealth.org (R.B.); 3 Department of Neurobiology, Barrow Neurological Institute, Phoenix, AZ 85013, USA; 4 Gregory W. Fulton ALS and Neuromuscular Disease Center, Barrow Neurological Institute, Phoenix, AZ 85013, USA; Shafeeq.Ladha@DignityHealth.org

**Keywords:** ALS, relaxivity contrast imaging, TRATE, perfusion MRI, muscle myofiber

## Abstract

(1) Background: This work characterizes the sensitivity of magnetic resonance-based Relaxivity Contrast Imaging (RCI) to Amyotrophic Lateral Sclerosis (ALS)-induced changes in myofiber microstructure. Transverse Relaxivity at Tracer Equilibrium (TRATE), an RCI-based parameter, was evaluated in the lower extremities of ALS patients and healthy subjects. (2) Methods: In this IRB-approved study, 23 subjects (12 ALS patients and 11 healthy controls) were scanned at 3T (Philips, The Netherlands). RCI data were obtained during injection of a gadolinium-based contrast agent. TRATE, fat fraction and T_2_ measures, were compared in five muscle groups of the calf muscle, between ALS and control populations. TRATE was also evaluated longitudinally (baseline and 6 months) and was compared to clinical measures, namely ALS Functional Rating Scale (ALSFRS-R) and Hand-Held Dynamometry (HHD), in a subset of the ALS population. (3) Results: TRATE was significantly lower (*p* < 0.001) in ALS-affected muscle than in healthy muscle in all muscle groups. Fat fraction differences between ALS and healthy muscle were statistically significant for the tibialis anterior (*p* = 0.01), tibialis posterior (*p* = 0.004), and peroneus longus (*p* = 0.02) muscle groups but were not statistically significant for the medial (*p* = 0.07) and lateral gastrocnemius (*p* = 0.06) muscles. T_2_ differences between ALS and healthy muscle were statistically significant for the tibialis anterior (*p* = 0.004), peroneus longus (*p* = 0.004) and lateral gastrocnemius (*p* = 0.03) muscle groups but were not statistically significant for the tibialis posterior (*p* = 0.06) and medial gastrocnemius (*p* = 0.07) muscles. Longitudinally, TRATE, averaged over all patients, decreased by 28 ± 16% in the tibialis anterior, 47 ± 18% in the peroneus longus, 25 ± 19% in the tibialis posterior, 29 ± 14% in the medial gastrocnemius and 35 ± 18% in the lateral gastrocnemius muscles between two timepoints. ALSFRS-R scores were stable in two of four ALS patients. HHD scores decreased in three of four ALS patients. (4) Conclusion: RCI-based TRATE was shown to consistently differentiate ALS-affected muscle from healthy muscle and also provide a quantitative measure of longitudinal muscle degeneration.

## 1. Introduction

Amyotrophic lateral sclerosis (ALS) is a neurodegenerative disease characterized by the progressive death of upper motor neurons (UMNs) of the primary motor cortex and corticospinal tract (CST), in conjunction with lower motor neurons (LMNs) associated with the anterior horns. Despite the recognition of UMN and LMN involvement as a characteristic signature, the mean diagnostic delay among ALS patients is around 12 months, primarily due to patients being misdiagnosed with more common diseases that might mimic the early stages of ALS [1]. The long diagnostic delay underscores the dearth of robust and sensitive clinical biomarkers in ALS. Clinical indicators of disease status, such as Revised ALS Functional Rating Scale (ALSFRS-R), electromyography (EMG), and muscle strength tests, may be confounded by inter-rater variability and/or low sensitivity to ALS; these factors are further compounded by the clinical heterogeneity of disease onset and progression. The efficacy of ALSFRS-R as a measure of clinical outcome can also be affected by the underreporting of functional impairment severity [2]. Hence, there is an urgent need to establish robust, non-invasive, and quantitative biomarkers that can serve as early and specific diagnostic and prognostic indicators of disease. 

Although current literature on motor neuron dysfunction in ALS is extensive, there is a continuing debate about whether motor neuron death is of a forward (UMN spreading to LMN) or backward (LMN spreading to UMN) nature [3]. Irrespective of the progression pathway, neuromuscular junction degeneration leads to skeletal muscle denervation and is known to accompany clinical symptom onset. Symptomatically, around 75% of ALS patients present limb muscle weakness, while others present a bulbar onset [4,5].

To date, the clinical role of magnetic resonance imaging (MRI) in ALS has primarily been limited to exclusion of other neurodegenerative diseases that present similar symptoms to ALS [6]. Advanced quantitative imaging methods, while promising, have predominantly focused on evaluating singular pathologic characteristics, such as the disrupted fiber tracks of UMNs [7,8]. Clinically, LMN dysfunction is primarily assessed using electromyography [9], electrical impedance myography [10,11], muscle ultrasonography [12] or muscle biopsies [13]. Previous imaging studies have employed semi-quantitative or quantitative approaches to characterize the LMN pathways of ALS disease progression [10,11,12]. One of the earliest studies of ALS muscle demonstrated that while edema regions exhibit relative T_1_ and T_2_ signal increases, fatty infiltration causes relative T_1_ to decrease and relative T_2_ to increase [11]. Another study used the relative T_2_ signal approach, wherein the authors additionally implemented a whole-body imaging protocol for their study [14]. They demonstrated that for ALS patients with bulbar, upper extremity, or lower extremity onset, the affected muscle regions exhibited increased relative T_2_ signal when compared with healthy muscle. More recently, diffusion-weighted imaging (DWI) has been added to this whole-body approach [14] and used to longitudinally evaluate patients over 12 months. The authors concluded that relative T_2_ signal, in comparison with DWI, was most effective at detecting longitudinal changes in leg muscle groups. A more quantitative approach was implemented when evaluating skeletal muscle differences in spinal and bulbar muscular atrophy (SBMA) and ALS patient populations when compared with healthy controls [15]. Fatty infiltration due to atrophy occurring from muscle denervation was quantified using m-Dixon-based fat fraction measures, and a semi-quantitative short tau inversion recovery (STIR) imaging approach was employed to evaluate edema arising from denervation in thigh, calf and tongue muscles. The fat fraction measures were more sensitive to differentiating SBMA muscle from healthy muscle, while ALS muscles were better identified using the STIR-based approach. 

While longitudinal studies have established the potential for muscle imaging to interrogate motor neuron disease progression using relative signal changes and quantitative measures of fatty infiltration [16], there remains a need for robust, quantitative, and sensitive imaging biomarkers to characterize LMN dysfunction, and in particular, ones that interrogate ALS-associated myofiber pathology. Biomarkers that are sensitive to myofiber architecture could enable more robust detection of disease progression and therapy response, as compared with downstream and indirect surrogates of disease status, such as edema. Contrast-enhanced MRI techniques have been successfully used to characterize tissue pathophysiology. We recently showed that, by simultaneously quantifying T_1_ and T_2_^*^ changes associated with the dynamic contrast agent passage, a unique parameter that reflects cellular microstructure can be quantified—a technique previously termed relaxivity contrast imaging (RCI) [17]. In particular, the contrast agent’s transverse relaxivity at tracer equilibrium, or TRATE, is an RCI parameter that is shown to be predominantly sensitive to cellular microstructure [18]. Given the microstructural changes in muscle myofibers that accompany, and potentially precede, muscle degeneration and atrophy, the purpose of this study is to provide the first evaluation of TRATE as a quantitative, noninvasive muscle imaging biomarker for ALS characterization and progression.

## 2. Materials and Methods

### 2.1. Patient Cohort

This study was approved by our institutional review board with informed consent obtained from each participant. The study group consisted of healthy control (12 participants, mean age: 51 ± 18 years, 8 female, 4 male) and ALS patient (14 participants, mean age: 65 ± 8 years, 7 female, 7 male) cohorts. All ALS patients included were diagnosed based on the modified El Escorial criteria [19], with time since diagnosis less than 24 months. All study participants underwent an MRI exam of the lower extremities. A subset of the ALS patient group (6 participants, mean age: 68 ± 6 years) underwent a second MRI exam after an average duration of 6 months to record longitudinal changes. In addition, in the ALS patients, reference clinical measures, namely hand-held dynamometry (HHD) of the hip flexors, knee flexors, knee extensors and ankle dorsiflexors and the ALSFRS-R, were performed before each imaging time point. Global function and lower-limb function were assessed from the ALSFRS-R scores. The ALSFRS-R_total_ score was obtained by adding the individual score responses in the questionnaire. The ALSFRS-R_LL_ was obtained by adding the individual score responses to lower-limb function in the questionnaire. Quantitative strength testing primarily assays the peripheral motor component of ALS and was measured using HHD as outlined in previous literature [20]. The lower limb HHD scores (HHD_total-left_ and HHD_total-right_) were obtained as the sum of HHD scores for left and right hip-flexion, knee-flexion, knee-extension, and ankle dorsiflexion.

### 2.2. Image Acquisition

All study participants underwent MRI on a 3T Ingenia (Philips, The Netherlands) scanner using both the FlexCoverage anterior and posterior body coils (Philips, The Netherlands) across both lower extremities (left and right calf muscle region). The participants were scanned feet first in a supine manner.

The study protocol consisted of an anatomical T_1_-weighted sequence, a multi-point mDixon Quant scan to obtain measures of fat fraction in the calf muscle, a multishot turbo spin echo (TSE) sequence to obtain T_2_ maps, a variable flip angle (VFA) approach to measure baseline T_1_ maps, and a multi-echo gradient echo (GRE) sequence to obtain the pre- and post-contrast measures of the transverse relaxation rate (R_2_^*^). In addition, a dual gradient echo dynamic scan was performed during the injection of a gadolinium-based contrast agent (Gadavist; 0.1 mmol/kg) to collect RCI data, enabling the quantification of contrast agent concentration and contrast agent T_2_^*^ relaxivity. The contrast agent was injected at the rate of 2 mL/s after acquiring 90 s of baseline data using a power injector followed by a saline flush. The scan parameters are provided in Table 1. The total scan time was approximately 32 min per participant. 

### 2.3. Image Analysis

Image analysis was performed using an in-house developed code in MATLAB (MathWorks Inc., Natick, MA, USA). All data were registered to the T_1_-weighted anatomical data using FLIRT (FSL) [21] with 12 degrees of freedom. Muscle groups from the anterior (Tibialis Anterior (TA), and Peroneus Longus (PL)) and posterior (Tibialis Posterior (TP), Lateral Gastrocnemius (LG), and Medial Gastrocnemius (MG)) regions of the calf were manually identified for postprocessing and analysis, on a slice-by-slice basis, by a scientific researcher (SR) with 6 years of MRI experience. TRATE [18] values were computed as the ratio of ${\Delta R}_{2}^{*}/C_{t}$ at contrast agent equilibrium (last 10 time points in the dynamic series), where C_t_ is the contrast agent concentration, computed as $\Delta R_{1}/r_{1}$. ${\Delta R}_{2}^{*}$ maps were calculated from the dual echo dynamic data as previously described [22]. Pre-contrast T_1_ maps were obtained using the variable flip angle approach [23]. TRATE estimates in the 5 muscle ROIs were compared with fat fraction (generated on scanner host by Philips post-processing) and T_2_ values (generated on scanner host by Philips post-processing) between ALS patients and healthy controls. Longitudinal TRATE estimates were also compared with ALSFRS-R (total and lower limb) and HHD (lower limb) scores among the ALS population. 

### 2.4. Statistical Analysis

All statistical analysis was performed in MATLAB (MathWorks Inc., Natick, MA, USA). TRATE, fat fraction and T_2_ estimates were compared between ALS-affected muscle and healthy control muscle using a paired t-test for each muscle group. The normality of the ROI-based population data was determined using the Lilliefors and Anderson-Darling tests. *p* values less than 0.05 were considered significant (two-tailed). Statistical analysis was not performed on the longitudinal data due to the limited number of available datasets (N = 4).

## 3. Results

### 3.1. Comparative Analysis

In the ALS cohort, two datasets were excluded due to a reconstruction error (n = 1) and incorrect imaging protocol (n = 1). Thus, for the comparative analysis with healthy controls, single time-point data were used from 12 ALS patients. 

**Figure 1:**
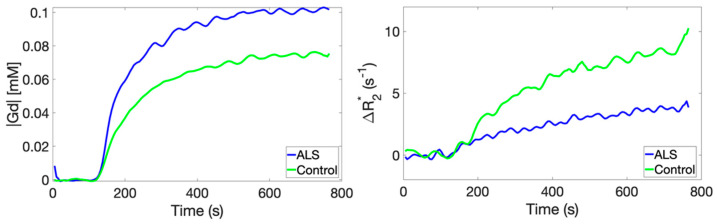
A comparison of time-dependent CA concentration curves (**left**) and transverse relaxation rate changes (**right**) obtained in the tibialis anterior muscle compartment of an ALS patient and a healthy control.

Example C_t_(t) and ∆R_2_*(t) time curves in the tibialis anterior muscle of a representative ALS patient and healthy control are shown in Figure 1. During contrast agent equilibrium, C_t_(t) uptake in ALS muscle was on average 0.11 ± 0.001 mM, and C_t_(t) uptake in healthy muscle was on average 0.08 ± 0.001 mM, which represents a 38% difference. During the contrast agent equilibrium, ∆R_2_*(t) in ALS muscle was on average 3.8 ± 0.25 s^−1^, and ∆R_2_*(t) in healthy muscle was on average 8.9 ± 0.78 s^−1^, which represents a 57% difference.

**Figure 2:**
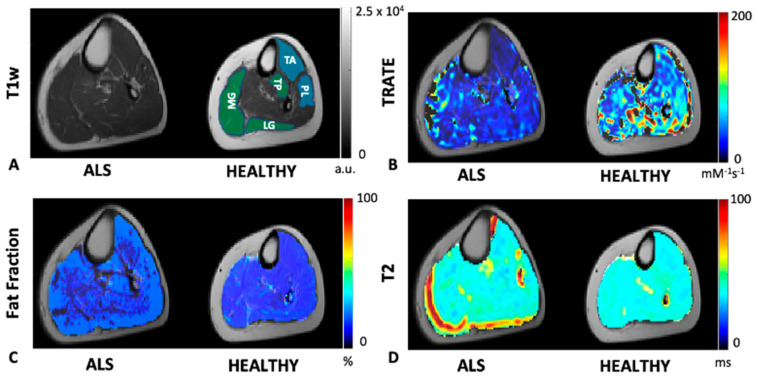
An example axial slice of T1w calf image is shown in (**A**) for an ALS patient and a healthy control. The control slice is highlighted with an anterior (blue) compartment comprising of the tibialis anterior (TA) and peroneus longus (PL) muscle ROIs and posterior (green) compartment comprising of the tibialis posterior (TP), medial and lateral gastrocnemius (MG and LG, respectively) muscle ROIs. TRATE, Fat Fraction and T2 maps were compared between the ALS and healthy control populations as shown in (**B**–**D**), respectively. TRATE was observed to be lower in ALS-affected muscle. While in some datasets obtained, fatty infiltration in ALS-affected muscle was observed, the dataset shown above is an example observation of similar fat content in healthy and ALS-affected muscle. In accordance with existing muscle imaging studies, the T2 map showed sporadic elevated regions in ALS muscle, but these changes were minor when compared with changes in TRATE measures.

A comparison of TRATE, fat fraction, and T_2_ maps between a healthy control and an ALS patient are illustrated in Figure 2. Across multiple muscle groups, TRATE was found to be consistently lower in ALS patients when compared with healthy controls. TRATE was lower on average in ALS-affected muscle by 54 ± 10%. Fat fraction and T_2_ values were increased among ALS patients by an average of 39 ± 26% and 18 ± 11%, respectively, when compared with healthy controls. Summary statistics of TRATE values in ALS patients and healthy controls are provided in Table 2. Across all the muscle group studies, the average TRATE was 82.15 ± 14.65 mM^−1^s^−1^ in healthy controls and 46.7 ± 9.5 mM^−1^s^−1^ in ALS patients.

**Figure 3:**
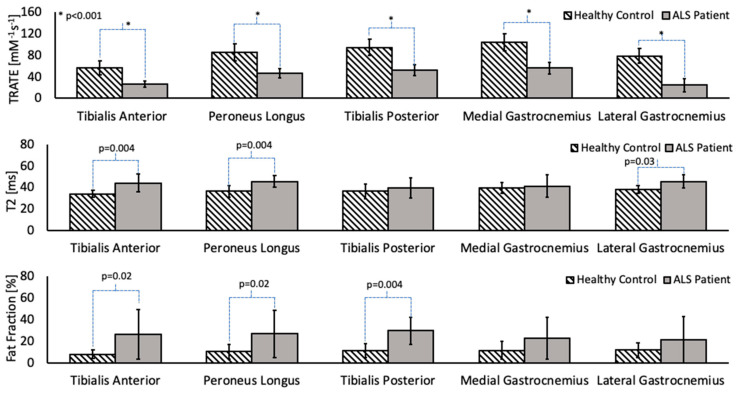
Group-wise analysis of TRATE, fat fraction and T2 values in cohorts of ALS and healthy subjects. TRATE was the only parameter to show statistically significant (*p* < 0.05) differences between patients and controls across all muscles studied.

Across patients, the differences in TRATE, fat fraction and T_2_ values between affected and healthy muscle are highlighted in Figure 3 for the tibialis anterior, tibialis posterior, peroneus longus, lateral head of the gastrocnemius and medial head of the gastrocnemius muscles. On average, the T_2_ and fat fraction values in ALS-affected muscle were found to be higher than in healthy muscle; however, there were muscle groups in multiple patients that did not exhibit a perceptibly higher fat fraction or T_2_, such as the example shown in Figure 2. A significant decrease in TRATE was observed across all muscle groups (*p* < 0.0001) in the ALS patients when compared with healthy control muscle groups. The fat fraction differences between ALS-affected and healthy muscle in the medial and lateral gastrocnemius muscle were not significantly different (*p* = 0.07 and *p* = 0.06, respectively) but were observed to be significantly different in the tibialis anterior (*p* = 0.01), tibialis posterior (*p* = 0.004), and peroneus longus (*p* = 0.02) muscle groups. The T_2_ differences between ALS-affected and healthy muscle in the tibialis posterior and medial gastrocnemius muscle were not statistically significant (*p* = 0.06 and *p* = 0.07, respectively) but were observed to be significant in the tibialis anterior (*p* = 0.004), peroneus longus (*p* = 0.004) and lateral gastrocnemius muscle groups (*p* = 0.03).

### 3.2. Longitudinal Analysis

A total of six ALS patients were part of the longitudinal imaging study. Two datasets were excluded due to a reconstruction error (n = 1) and incorrect imaging protocol (n = 1), resulting in four datasets for the longitudinal analysis.

**Figure 4:**
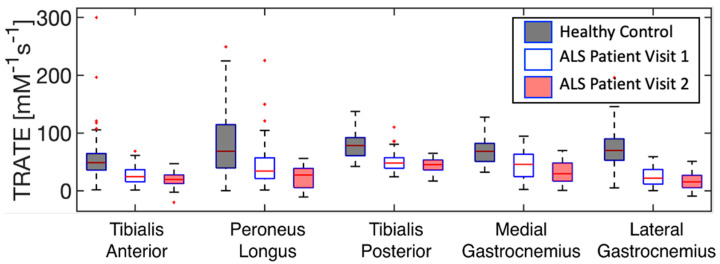
Evolution of TRATE measurements over time in a representative dataset are presented as boxplots for each muscle group. TRATE reduction between visits 1 & 2 was observed among all ALS affected muscle groups.

**Figure 5:**
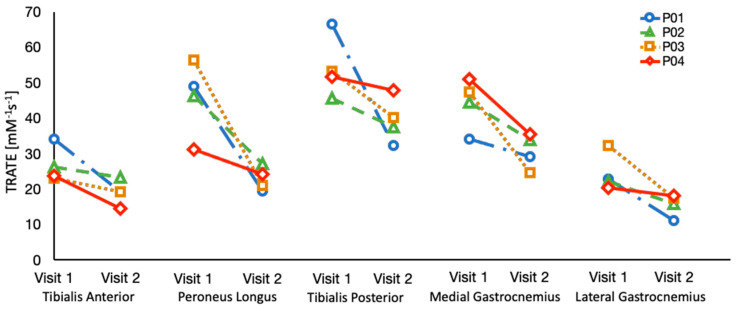
TRATE data was collected over an interval of 6 months in four ALS patients. TRATE values consistently decreased between the two time points, suggestive of progressive disease.

The evolution of TRATE measurements in a representative ALS dataset over two timepoints and TRATE measurements in a representative healthy control dataset at a single timepoint are presented as boxplots for each muscle group in Figure 4. Across all muscle groups, TRATE decreased between the two imaging time points. Furthermore, TRATE values between visits were both lower than those found in healthy controls across all muscle groups. Figure 5 highlights the longitudinal evolution of average TRATE measures for each muscle group. Table 3 compares TRATE (averaged over all muscles) measures with ALSFRS-R and HHD scores. ALSFRS-R total scores decreased on average at the rate of 0.8%/month, and ALSFRS-R LL scores decreased on average at a rate close to 2%/month. HHD scores decreased on average at the rate of a little over 2%/month. Average TRATE decreased at a rate close to 4%/month. TRATE measures for each muscle ROI decreased on average as follows: tibialis anterior at the rate of 4%/month, peroneus longus at the rate of 8%/month, tibialis posterior at the rate of 3%/month, medial gastrocnemius at the rate of 5%/month and lateral gastrocnemius at the rate of 5%/month. While ALSFRS-R and HHD scores were expected to decrease between the two visits, confounding results were observed while recording both clinical measures. Two patients did not exhibit any difference in ALSFRS-R scores (total and lower limb) between the two visits (ALSFRS-R_total_ = 42, ALSFRS-R_LL_ = 7 and ALSFRS-R_total_ = 39, ALSFRS-R_LL_ = 6), while TRATE was found to decrease in each patient between the two visits. When averaged over all patients, TRATE decreased by 28 ± 16% in the tibialis anterior, 47 ± 18% in the peroneus longus, 25 ± 19% in the tibialis posterior, 29 ± 14% in the medial gastrocnemius and 35 ± 18% in the lateral gastrocnemius muscles between visits. Individual ALSFRS-R scores for the longitudinal data are presented in Supplementary Digital Content SDC1. Two patients scored paradoxically higher on the muscle strength test during visit 2 (HHD_total, left_ = 140 lbs, HHD_total, right_ = 139.3 lbs and HHD_total, left_ = 98.2 lbs) compared with visit 1 (HHD_total, left_ = 129.8 lbs, HHD_total, right_ = 135.9 lbs and HHD_total, left_ = 78.4 lbs), although for one patient, this was attributed to recorded symptom onset and progression in the lower right extremity. The individual HHD scores for both visits are outlined in Supplementary Digital Content SDC2.

## 4. Discussion

In this work, we have demonstrated a quantitative approach to consistently differentiate ALS-affected calf muscle from healthy muscle by comparing TRATE, fat fraction, and relaxometry derived T_2_ values. A major highlight of this work is that among all quantitative imaging and clinical metrics, only TRATE demonstrated consistent changes between (a) ALS and healthy muscle, and (b) the two timepoints of data acquisition for every single ALS patient. These findings support the hypothesis that RCI is sensitive to ALS-induced aberrations in muscle myofiber architectural features (e.g., reduced fiber diameter, density, atypia).

The use of a multi-echo dynamic gradient echo sequence enabled the simultaneous quantification of T_1_ and T_2_^*^ relaxation times [17,24]. The T_1_ changes enable quantification of the local contrast agent concentration. The T_2_^*^ changes depend on the contrast agent concentration but, more importantly, reflect contrast-agent-induced magnetic field perturbations resulting from the compartmentalization of the agent within tissue compartments (e.g., extracellular space surrounding myofibers). A fundamental characteristic of the resulting perturbations is their dependence on the geometry of the compartment containing the contrast agent. In muscle, the contrast agent can leak out of blood vessels and distribute around muscle fibers. The microscopic interaction of contrast agent and water in each compartment leads to the observed T_1_ changes. While this interaction will shorten local T_2_ values, the more predominant effect on the observed T_2_^*^ changes originates from the mesoscopic magnetic field perturbations that occur as the contrast agent is compartmentalized around the myofibers. Accordingly, the differences in the dynamic ∆R_2_* and ∆R_1_, from which C_t_ is computed, can be attributed to their dissimilar contrast mechanisms. In this patient cohort, relatively minor changes to C_t_ were observed between ALS and healthy controls, indicating a larger influence of ∆R_2_* on TRATE. The muscle-associated ∆R_2_* changes reflect the local fiber properties, such as fiber density, geometry, organization, heterogeneity and size. Increased muscle atrophy as a result of the muscle fiber denervation process with ALS disease progression is expected to influence the muscle fiber density and diameter, which our prior computation studies predict should lead to reduced TRATE values [18], as observed herein when comparing healthy versus ALS muscle and a given ALS muscle across time. TRATE is also inversely affected by contrast agent concentration. However, we observed that the contrast agent concentration evolution over time was comparable in both healthy and ALS-affected muscle and hence had minimal effect on TRATE. This also suggests that DCE-MRI alone may not be able to detect ALS-induced changes in muscle microstructure. 

Another important observation was that relative change in TRATE, fat fraction and T_2_ between ALS and healthy muscles varied across different muscle groups. This was consistent with the heterogeneity in relative T_2_ measures observed across different muscle groups, as reported previously [15]. The heterogeneity could be attributed to varying degrees of muscle atrophy within individual ALS patients and the respective contrast mechanisms of each imaging approach. It has been hypothesized that some of the more active muscle groups such as the Tibialis Anterior have a higher atrophy rate [25]. At the time of imaging, the extent of muscle atrophy for each muscle group could be different due to the time elapsed between disease onset and imaging timepoint(s) in the study population. Additionally, this phenomenon could also be influenced by a multitude of other factors, such as age, exercise, muscle fiber orientation, and muscle volume. 

This work also provides a preliminary evaluation of RCI-based TRATE as a biomarker of ALS disease progression. TRATE consistently declined in ALS patient lower extremity muscles, while the clinical measures remained stable or even improved slightly. TRATE reduced by a greater amount over time in the peroneus longus muscles than the tibialis anterior muscles. The tibialis anterior changes could have occurred earlier in the disease progression, while the larger peroneus longus changes could have occurred during the imaging time points of the study. Future studies will evaluate earlier stages of the disease to more systematically characterize longitudinal TRATE changes in each muscle group. While it is not uncommon to observe unchanged ALSFRS-R scores in patients with slowly progressing ALS after a period of 6 months, the results suggest that TRATE may outperform clinical measures, which can have significant variability, as a measure of disease progression. It has to be noted that only a subset of the ALS patient population was scanned at the 6-month timepoint. If a larger data set can confirm this, TRATE may become a useful measure in clinical trials to detect treatment effects better than some clinical measures. 

There are a few limitations to this study. Primarily, the patient and control population sizes are small. However, future longitudinal studies will aim to increase patient population size. Secondly, the muscle regions of interest were manually segmented and could likely benefit from automated/semi-automated muscle segmentation tools for improved consistency [26,27,28,29]. Due to scan times being influenced by the ability of ALS patients to lie still in the MRI scanner, additional limits had to be imposed on the image quality with respect to spatial and temporal resolution, scan time for each protocol and number of scans. Thirdly, longitudinal measurement of TRATE in limb muscle does not inform us of upper motor neuron dysfunction in ALS, which occurs simultaneously with lower motor neuron dysfunction and also affects the clinical measures we are comparing our imaging markers with.

Further work is required to validate RCI’s potential as a biomarker for patients with ALS. To systematically characterize differences between healthy and ALS muscle, it would be informative to characterize TRATE across relevant age groups. Additional information could be obtained from an RCI performance comparison between sporadic ALS and familial forms of ALS. Some ALS gene mutations can impact muscle, which may more dramatically impact TRATE measures. While this study focused on imaging muscles in the leg, it will be important evaluate RCI’s potential in other relevant muscle groups (e.g., bulbar imaging). To establish its utility as a biomarker, the repeatability of RCI needs to be established, and its sensitivity to early therapeutic response should be compared with other markers of myofiber microstructure (e.g., electromyography, electric impendence myography and muscle ultrasonography [9,12,30,31,32]). Preclinical and computational studies could also shed light on the myofiber microstructural features that contribute to the observed TRATE changes and help guide future clinical interpretation.

## 5. Conclusions

In this work, RCI was introduced as new quantitative approach to evaluate ALS-afflicted myofiber architectural changes across multiple calf muscle groups; additionally, the potential of this biomarker to assess longitudinal muscle degeneration was demonstrated. RCI-derived TRATE measures were more consistent compared with other imaging measures in identifying diseased muscle and were more sensitive to longitudinal myofiber changes when compared with standard clinical measures obtained over the same period. A multi-parametric approach integrating muscle imaging techniques characterizing LMN involvement and neuroimaging techniques characterizing UMN involvement could be essential to developing robust quantitative imaging biomarkers for ALS disease characterization and evaluating longitudinal treatment response.

## Figures and Tables

**Table 1:** List of select MRI acquisition parameters for each protocol of the exam.

	Dual Echo Time Series	Multi-Echo	T1 Map (VFA)	mDixon	T2 Map	T1w TSE
TR [ms]	21	34	7.7	8.5	3605	598
TE(s) [ms]	[1.06, 20.0]	[4.9, 11.4, 17.9, 24.4, 30.9]	4.6	1.4	[12.0, 18.1, 24.2, 30.3, 36.4, 42.5]	8
Flip Angle(s) [^o^]	25	25	[20,18, 16, 14, 12, 10, 8, 6, 4, 2]	3	90	90
Acq. Resolution [mm^2^]	3.0 × 3.0	1.5 × 1.5	2.5 × 2.5	1.3 × 1.3	3.4 × 3.4	1.0 × 1.0
Slice Thickness [mm]	4.0	4.0	4.0	4.0	5.0	5.0
# Dynamics	150	-	-	-	-	-
# Echoes	2	5	1	6	6	1
TSE factor	-	-	-	-	6	5
SENSE	3.5 (RL), 1.5 (AP)	2 (AP), 1.25 (FH)	2 (AP)	-	2 (AP)	-
FOV [mm^3^]	300 × 230 × 248	300 × 230 × 248	300 × 230 × 248	300 × 230 × 195	300 × 230 × 248	300 × 230 × 248
Scan Time [mm:ss]	12:55	01:42	03:36	01:12	04:19	04:51
Imaging Metric	TRATE [mM^−1^s^−1^] ∆R_2_*(t) [s^−1^]	T_2_* [ms]	T_1_ [ms]	Fat Fraction [%]	T_2_ [ms]	Signal Magnitude [a.u]

**Table 2:** Range of TRATE [mM^−1^s^−1^] values in ALS and Healthy Control populations.

Muscle Group	Mean (SD)		95% Confidence Interval	
	ALS Patient	Healthy Control	ALS Patient	Healthy Control
Tibialis Anterior	27.67 (5.54)	71.09 (13.52)	[24.7, 30.7]	[62.5, 79.7]
Peroneus Longus	52.75 (8.76)	80.50 (15.52)	[48.0, 57.9]	[70.6, 90.4]
Tibialis Posterior	43.94 (10.02)	83.80 (15.14)	[38.5, 49.4]	[74.2, 93.4]
Medial Gastrocnemius	58.72 (10.49)	89.56 (15.35)	[53.0, 64.4]	[79.8, 99.3]
Lateral Gastrocnemius	50.39 (12.67)	85.81 (13.71)	[43.5, 57.3]	[77.1, 94.5]

**Table 3:** Longitudinal (N = 4) data comparison of clinical scores (ALSFRS-R, HHD) and Imaging metric (TRATE) as percent change in recorded value between visits.

Parameters	(Visit 2 − Visit 1)/Visit 1 [%]
ALSFRS-R Total [a.u.]	−4.70 *
ALSFRS-R Lower Limb [a.u.]	−13.04 *
HHD left [lbs]	−12.18 *
HHD Right [lbs]	−16.42 *
TRATE [mM^−1^ s^−1^]	−21.62 *

* Negative change is resulting from decrease in recorded metric between visits.

## Data Availability

The data presented in this study are available on request from the corresponding author.

## Supplementary Materials

The following are available online at https://www.mdpi.com/article/10.3390/tomography7020015/s1, Table S1: ALSFRS-R Scores and corresponding TRATE measures for each longitudinal dataset, Table S2: HHD Scores [lbs.] for each longitudinal dataset.
