# Supplementary material for: Evaluation of Amyotrophic Lateral Sclerosis-Induced Muscle Degeneration Using Magnetic Resonance-Based Relaxivity Contrast Imaging (RCI)
Source: Tomography. 2021 May 5;7(2):169–79. doi: 10.3390/tomography7020015 (PMC8162571; doi:10.3390/tomography7020015)
Supplement: Supplementary file 1 [file tomography-07-00015-s001.zip › ClinicalALS_SupplementaryData/ClinicalALS_SDC1.pdf]

Supplementary Digital Content SDC1: ALSFRS-R Scores and corresponding TRATE measures for each longitudinal dataset

Longitudinal Data	Visit 1			Visit 2		
	ALSFRS-R Total	ALSFRS-R LL	TRATE [$mM^{-1}s^{-1}$]	ALSFRS-R Total	ALSFRS-R LL	TRATE [$mM^{-1}s^{-1}$]
**P1**	42	7	92.06	42	7	79.95
**P2**	39	6	69.38	39	6	57.41
**P3**	36	4	73.33	31	3	53.40
**P4**	32	6	63.35	30	4	42.93
