# Supplementary material for: Evaluation of Amyotrophic Lateral Sclerosis-Induced Muscle Degeneration Using Magnetic Resonance-Based Relaxivity Contrast Imaging (RCI)
Source: Tomography. 2021 May 5;7(2):169–79. doi: 10.3390/tomography7020015 (PMC8162571; doi:10.3390/tomography7020015)
Supplement: Supplementary file 1 [file tomography-07-00015-s001.zip › ClinicalALS_SupplementaryData/ClinicalALS_SDC2.pdf]

Supplementary Digital Content SDC2: HHD Scores [lbs.] for each longitudinal dataset

ALS Patient#		Hip Flexion		Knee Flexion		Knee Extension		Ankle Dorsiflexion		Total Lower Limb	
		Left	Right	Left	Right	Left	Right	Left	Righ	Left	Right
01	Visit 1 (V1)	31.40	29.90	26.60	31.90	26.10	29.10	45.70	45.00	**129.80**	**135.90**
	Visit 2 (V2)	30.60	28.70	31.60	29.10	42.40	36.90	35.40	44.60	**140.00**	**139.30**
	V1 - V2	**0.80**	**1.20**	**-5.00**	**2.80**	**-16.30**	**-7.80**	**10.30**	**0.40**	**-10.20**	**-3.40**
02	Visit 1 (V1)	32.50	15.80	21.90	14.70	29.70	16.90	0.00	0.00	**84.10**	**47.40**
	Visit 2 (V2)	7.60	7.30	7.40	4.80	11.70	5.20	0.00	0.00	**26.70**	**17.30**
	V1 - V2	**24.90**	**8.50**	**14.50**	**9.90**	**18.00**	**11.70**	**0.00**	**0.00**	**57.40**	**30.10**
03	Visit 1 (V1)	22.60	24.30	15.60	12.80	12.80	17.10	27.40	22.20	**78.40**	**76.40**
	Visit 2 (V2)	25.10	21.70	15.30	16.00	33.90	28.40	23.90	0.00	**98.20**	**66.10**
	V1 - V2	**-2.50**	**2.60**	**0.30**	**-3.20**	**-21.10**	**-11.30**	**3.50**	**22.20**	**-19.80**	**10.30**
04	Visit 1 (V1)	15.50	6.00	10.90	10.40	16.30	10.70	0.00	0.00	**42.70**	**27.10**
	Visit 2 (V2)	14.30	6.00	7.00	7.00	8.00	4.00	0.00	0.00	**29.30**	**17.00**
	V1 - V2	**1.20**	**0.00**	**3.90**	**3.40**	**8.30**	**6.70**	**0.00**	**0.00**	**13.40**	**10.10**
